# Book Reviews

**Published:** 1916-02

**Authors:** 


### BOOK REVIEWS

**Books mentioned may be inspected at and ordered through this office. So far as possible, books received in any month will be reviewed in the issue of the second month following.**

---

**Automobile Repairing Made Easy.** Victor W. Page, M. E. Published by the Norman W. Henley Pub. Co., 132 Nassau Street, N. Y. 1060 pages, over 1000 specially made engravings, $3.00.

This book considers the various types of the component parts of automobiles in general, describes shop equipment, methods of demounting and assembling, etc. Its title should not be taken too literally by the amateur, possessed of a monkey wrench, screw driver and hammer, and who wishes merely to keep his own car in order. It is intended for the mechanic, professional or otherwise, who seriously intends to enter thoroughly into the subject. From this standpoint, the subject has been rendered as easy as possible. It includes, however, a good many subjects, such as carbon-removal, anti-freezing solutions, adjustment of spark plugs and the like, which appeal to the car owner who wishes to attend to certain details but who does not intend to equip a shop for all sorts of repair work. It also gives a practical understanding of the automobile, valuable to any owner who cannot always be personally attended by a mechanic and who needs some estimate of the value of services rendered.

---

**Transactions of the American Urological Association,** 14th Annual Meeting at Baltimore, April 13, 1915. 479 pages, illustrated. Publication Committee: Hugh Cabot, R. F. O'Neil, Geo. G. Smith.

This work is a symposium of the highest value. Two papers are of especial interest to the internist: Victor C. Pedersen's on Limitations of Functional Tests of the Kidneys, and Ernest F. King's report of a case of Alkaptonuria with references to literature.

---

**A Brief Bibliography** of Books Relating to the Latin-American Republics. Peter H. Goldsmith. Published by the McMillan Co., N. Y. 107 pages.

Brief reviews are given in many instances, to save time when the scope of the work or its importance might not be understood from the title. Invaluable for anyone attempting to make a study of our southern neighbors, from almost any standpoint. So far as our readers are concerned, we would particularly urge that anyone inclined to make investments, or to take up practice in the Spanish speaking countries of this continent, should read up thoroughly on the general economic, political and geographic topics.

---

**Introduction to the Study of Maya Hieroglyphs.** S. G. Morley. Bulletin No. 57 of the U. S. Bureau of Ethnology.

This treats almost entirely of the numeral signs and the wonderfully elaborate calendar. Otherwise, very little advance has been made toward deciphering the glyphs, and it is not even established how far they were ideographic and how far phonetic by the use of rebuses. Some general information is included. The brief description of the regular army and the raising of men for war is especially interesting at present.

---

**The Obstetric Quiz for Nurses,** Hilda Elizabeth Carlson, N. Y. Published by the Rebman Co., N. Y. 316 pages, $1.50.

A logical presentation of the various subjects of medicine needed for nursing, is arranged under questions in black letters. Thus, while literally a quiz compend, the book is really much more.

---

**Hospitals and the Law,** E. V. Mitchell, LL.B., University of S. Dakota. Published by the Rebman Co., N. Y. 178 pages, $1.75.

Rights and responsibilities, officials and attendants, administration and regulation, foundation and organization, remuneration and support are the main topics considered. A special chapter deals with military and naval hospitals. A table of cases cited will prove very useful in litigation, and the index allows one to refer to various problems that may arise. The value of such a work to any hospital, scarcely needs to be emphasized. Like the individual physician, the hospital bears an important relation to the community, one which imposes many duties, including those of a purely technical compliance with a multiplicity of laws and one which makes it vulnerable to attacks from various standpoints.

---

**Rockefeller Foundation, Annual Report 1913-14,** second edition.

This is largely an executive report but includes interesting descriptions of the work of the international commission in foreign lands, investigation of industrial conditions, mental hygiene, war relief, etc.

---

**Theory and Practice of Blood Letting,** Heinrich Stern, M. D., LL.D., N. Y. Published by the Rebman Co., N. Y. 264 pages, illustrated, $2.50.

While this work deals in an interesting way with ancient and mediaeval medical history, it is far more than a literary review of an extinct practice. Blood letting is a therapeutic measure approved by centuries of experience. While carried to an excess in the past it has, in recent times, been greatly neglected, partly from a reaction against such excess, partly because it involves a degree of manual skill a psychic element a little too surgical for the strict internist and not surgical enough for the surgeon who is essentially an operator. Besides, there has been lacking a practical critical guide, in accordance with modern scientific knowledge and the practical experience of living practitioners. This lack, Dr. Stern has supplied, with the broad and deep study which has characterized his research along various other lines.

---

**An Introduction to Bacteriology for Nurses,** Harry W. Carey, A. B., M. D., Troy, N. Y. Published by the F. A. Davis Co., Philadelphia. 144 pages, illustrated, $1.00.

If this book did nothing more than present to the nurse, in readable and understandable form, the general principles of teriology, infection, immunity, etc., it would serve a useful purpose. There is, however, an economic demand for office nurses and special assistants who must combine something of the practical knowledge of a technical assistant with the ordinary services of a nurse. Such positions are often highly desirable from the nurse's standpoint, and the surgeon, particularly, is inclined to offer them to nurses possessed of a moderate degree of technical skill who can be trained along special lines of interest. This book, therefore, fills a genuine want, and it has been well designed to meet its purpose.

---

**Speaking of Operations,** Irving S. Cobb. Published by the Geo. H. Doran Co., 38 W. 32d, N. Y. 64 pages, 50 cents.

This is, so to speak, a worm's-eye view of surgery. Our profession is made the butt of a good deal of good natured wit and we trust that all members who read the book will take it with equal good nature and may derive some lessons from it, especially with reference to the unnecessarily elaborate rituals which some men throw about their examinations and procedures. The author's attitude is illustrated in the following: "When I read in the medical journals that the eminent Dr. Somebody has succeeded in transferring the interior department of a pelican to a pointer pup and vice versa, with such success that the pup drowned while diving for minnows, and the pelican barked himself to death baying at the moon, I am interested but fail to see wherein the treatment of infantile paralysis has been materially advanced. On the other hand, I would rather the kind and gentle Belgian hare should be offered up as a sacrifice . . . . than that I should have something painful which can be avoided through making him a martyr." We are inclined to take this jest so seriously as to predict that the whole problem as to control of vivisection will be worked out on just about the lines suggested.

This book is accompanied with a report of a dinner tendered to Mr. Cobb, at the Waldorf-Astoria, April 25, 1915.

---

**39th Annual Report of the Buffalo Eye and Ear Infirmary,** for the year to Oct. 1, 1915.

As this pamphlet is already in the hands of the profession, we merely commend the excellent work of the institution.

---

**Saunders' Catalogue of Books;** Medicine, Surgery, Veterinary, Dentistry, Nursing. W. B. Saunders Co., W. Washington Square, Philadelphia.

This is a well illustrated descriptive work. We advise those who have not received it to apply to the firm.

---

**Annual Report of the Surgeon General of the Public Health Service, 1915.** Government Printing Office, Washington.

This is not merely a financial and executive report but includes a brief description of sanitary work along various lines. The needs of the service are stated to be: More commissioned officers, a moderate increase in the clerical force, additional appropriations for rural sanitary study, control of occupational diseases, of pollution of coastal waters, for the preparation of viruses, etc, for the segregation and care of lepers, and certain repairs and additions to buildings. There are few government services which repay their maintenance so directly and so well as this. It is perhaps especially necessary at this time, when there is so much discussion of military and naval preparedness, to emphasize the fact that this service should not only be adequately supported so far as its present functions and the immediately necessary extension of its usefulness are concerned, but that it should be brought up to the ideal maximum by increase of its powers and appropriations. Up to the point at which national supervision would duplicate or conflict with necessary local activities in regard to the public health, no expenditure of the public funds can produce a higher degree of efficiency than through the development of this service. If any special argument is needed to convince legislators who might be inclined to view the subject of preparedness in too narrow a spirit, it may be said that this service is so organized that, by comparatively slight additional training of its personnel, it could, in an emergency, be transferred bodily to the military and naval medical duties which are so important an item in war.

**Transactions of the Section on Obstetrics, Gynaecology and Abdominal Surgery** of the A. M. A. San Francisco Meeting, June 22-25, 1915. Reprinted from the Jour. of the A. M. A.

This is an illustrated, cloth bound volume of 250 pages containing papers of the highest merit, and is a most convenient form in which they may be preserved by those especially interested.

---

**Laboratory Methods,** with Special Reference to the Needs of the General Practitioner, by B. G. R. and E. G. C. Williams, with an introduction by Victor C. Vaughan, M. D., LL.D. Published by the C. V. Mosby Co., St. Louis. 3d edition, 43 engravings, 214 pages, $2.50.

We have previously reviewed the first two editions of this work. By its eminently practical adaptation of scientific truth to clinical medicine and the rare ability of its authors to gauge the time, apparatus and skill of the practicing physician along laboratory lines, it has made good. On this account the authors have been conservative in adding new material. Of the large number of tests proposed, since the appearance of the second edition, many have been tried out and abandoned; others require confirmation before they should be placed before the profession generally; others are too complicated; still others are obviously impracticable or based on false conceptions. The principal additions are: Elastic Tissue Staining; Salting-out Method for Tubercle Bacilli, An Invariable Blood Stain; A Dressing for Laboratory Tables.

---

**Diseases of the Skin,** Henry R. Hazen, A. B., M. D., Washington, D. C. Published by the C. V. Mosby Co., St. Louis. 233 illustrations, including 4 plates in color, 539 pages.

About a sixth of the book deals with general principles of anatomy and phsiology, diagnosis, treatment, hygiene, etc. The remainder is a classified discussion of the various lesions and diseases. Neoplasms are included, as they come under the general subject of dermatology and brief attention is given to the hair, nails and perspiration. The description and discussion of the diseases is clear and concise, and the illustrations, paper and print, are of the best.

---

**Zeitkinder,** by Dr. Henry Jones Mulford.

This book was reviewed in our last issue, but we wish to recur to the prologue in which Lucifer sends his imps to curse the baby about to be born. The author takes up an old superstition and turns it in the direction of reality by naming the imps: Ignorance, Selfishness, Doubt and Crime. One of the best methods of criticism of a book is the test of what ultimately holds the attention rather than the elements that first appeal to the mind as most excellent. In getting at the truth in the old superstitious notion a most valuable lesson is taught. We wish that this drama could be filmed and see no reason why it could not be. It not only affords many strong scenes, entertaining in themselves, but it is most distinctly a play with a purpose, a vital purpose and one that is sensibly brought to the mind of the reader.

